# Fluid Overload in Critically Ill Children

**DOI:** 10.3389/fped.2018.00306

**Published:** 2018-10-29

**Authors:** Rupesh Raina, Sidharth Kumar Sethi, Nikita Wadhwani, Meghana Vemuganti, Vinod Krishnappa, Shyam B. Bansal

**Affiliations:** ^1^Department of Nephrology, Akron Children's Hospital and Cleveland Clinic Akron General, Akron, OH, United States; ^2^Akron Nephrology Associates, Cleveland Clinic Akron General, Akron, OH, United States; ^3^Department of Nephrology, Kidney & Urology Institute, Medanta, The Medicity, Gurgaon, India; ^4^College of Medicine, Northeast Ohio Medical University, Rootstown, OH, United States; ^5^College of Graduate Studies, Northeast Ohio Medical University, Rootstown, OH, United States

**Keywords:** fluid overload, acute kidney injury, critical care, pediatric nephrology, intensive care

## Abstract

**Background:** A common practice in the management of critically ill patients is fluid resuscitation. An excessive administration of fluids can lead to an imbalance in fluid homeostasis and cause fluid overload (FO). In pediatric critical care patients, FO can lead to a multitude of adverse effects and increased risk of morbidity.

**Objectives:** To review the literature highlighting impact of FO on a multitude of outcomes in critically-ill children, causative vs. associative relationship of FO with critical illness and current pediatric fluid management guidelines.

**Data Sources:** A literature search was conducted using PubMed/Medline and Embase databases from the earliest available date until June 2017.

**Data Extraction:** Two authors independently reviewed the titles and abstracts of all articles which were assessed for inclusion. The manuscripts of studies deemed relevant to the objectives of this review were then retrieved and associated reference lists hand-searched.

**Data Synthesis:** Articles were segregated into various categories namely pathophysiology and sequelae of fluid overload, assessment techniques, epidemiology and fluid management. Each author reviewed the selected articles in categories assigned to them. All authors participated in the final review process.

**Conclusions:** Recent evidence has purported a relationship between mortality and FO, which can be validated by prospective RCTs (randomized controlled trials). The current literature demonstrates that “clinically significant” degree of FO could be below 10%. The lack of a standardized method to assess FB (fluid balance) and a universal definition of FO are issues that need to be addressed. To date, the impact of early goal directed therapy and utility of hemodynamic parameters in predicting fluid responsiveness remains underexplored in pediatric resuscitation.

## Introduction

In a critically ill patient, fluid balance is imperative in management and maintaining homeostasis. More often than not, patients are resuscitated with fluids to maintain adequate intravascular volume. A common issue seen in pediatric intensive care units is fluid imbalances and hemodynamic instability. Aggressive fluid administration can lead to fluid overload (FO), a condition in which there is a positive fluid balance in the patient. This phenomenon has been associated with a multitude of unfavorable effects and can further complicate the patient's condition. While FO itself is no indicator of mortality, the adverse effects of FO on an already at risk population puts them at an increased risk of morbidity and mortality. Research on this commonly practiced standard of care has shown that inundation is not always the best method for hemodynamic optimization. Current literature shows that there is a lack of a standard definition of fluid overload and a standard guideline by which to direct fluid therapy. The purpose of this study was to review the literature highlighting the impact of FO on a multitude of outcomes in critically-ill children, analyzing the causative vs. associative relationship of FO with critical illness, and addressing the current pediatric fluid management guidelines.

## Methods

**Figure 1:**
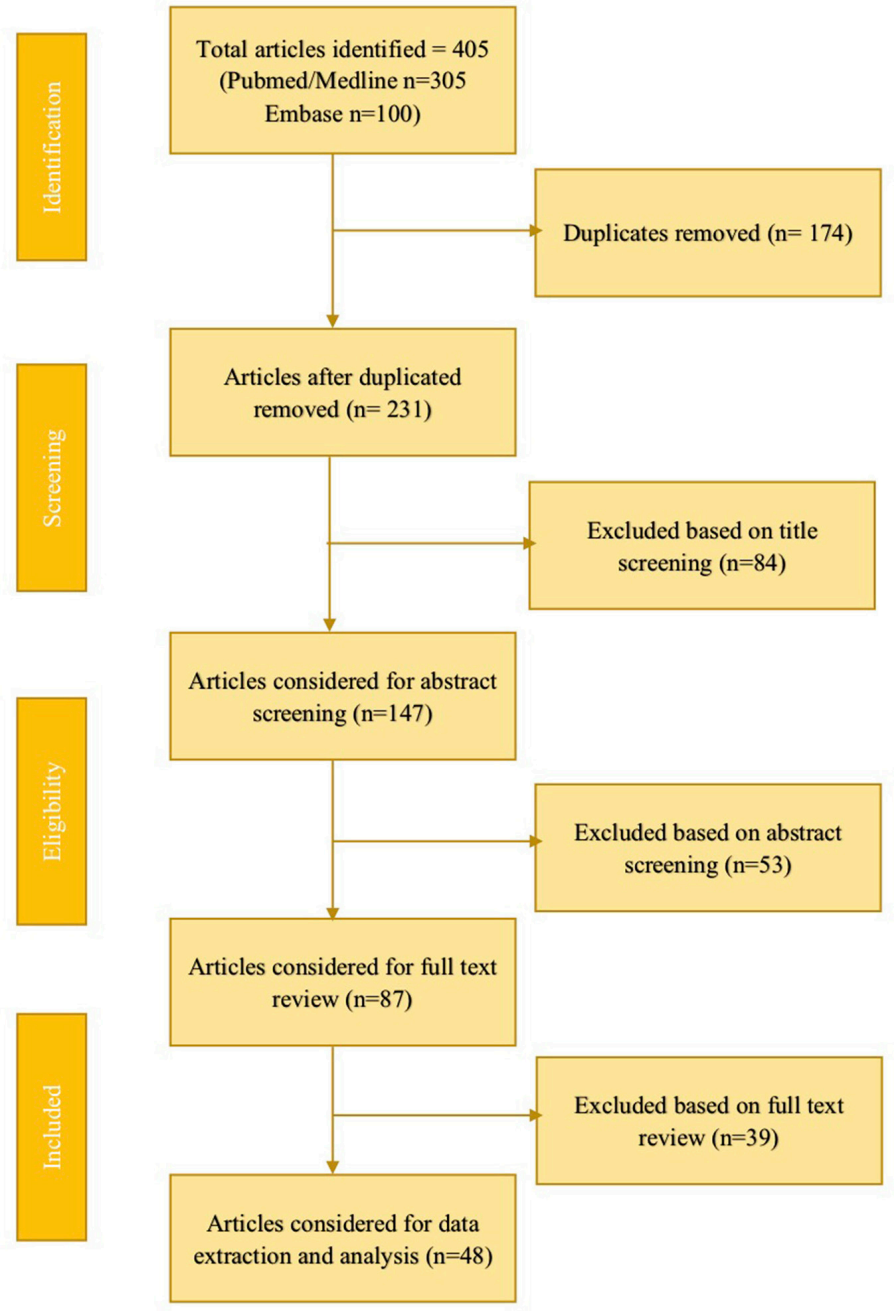
Summary of literature search.

The literature search was conducted using PubMed/Medline, and Embase databases for studies on fluid overload in pediatric critical care patients <18 years, from the earliest available date until June 2017. A total of 477 articles were obtained; after duplicate removal and review, 48 articles were selected for data extraction (Figure 1). The initial literature review consisted of two authors who independently reviewed the articles. The manuscripts of studies deemed relevant to the objectives of this review were then retrieved and associated reference lists hand-searched. Articles were categorized into FO associated with renal replacement therapy, extracorporeal membrane oxygenation, cardiac surgery, neonates, respiratory tract disease, and sepsis. All relevant publications were considered for data extraction. Tables were created summarizing the outcomes of the included studies.

## Pathophysiology of fluid overload

Current understanding of FO is contingent upon the revised Starling law which ascribes an important role to EGL (endothelial glycocalyx) in maintaining an intact vascular biology. EGL, a web of membrane-bound glycoproteins and proteoglycans on the luminal surface of endothelium, binds to plasma proteins and effectively excludes proteins from the sub-endothelial cleft, thereby resulting in a local oncotic gradient between plasma and cleft which opposes trans-capillary efflux (1). The law states that a constant oncotic gradient present along capillaries opposes pressure-mediated fluid efflux without ever causing fluid reabsorption from interstitium (except for vascular beds in renal tubules and intestines), and almost all vascular refilling from interstitium occurs via lymphatics (2). EGL breakdown can be triggered by various processes and stresses such as sepsis, surgery, postischemic states, and hyperglycemia (2–7). Once the EGL is damaged, fluid efflux becomes primarily dependent on capillary hydrostatic pressure and the manipulation of plasma oncotic pressure with colloids does not encourage any vascular refilling. In addition to fluid and protein extravasation, EGL disruption potentiates ongoing inflammatory responses by exposing endothelial cell-adhesion molecules and may account for pathologies with interstitial edema such as acute lung injury (4). In experimental and perioperative settings, volume expansion has been shown to hasten glycocalyx disintegration and reduce lymphatic drainage through release of natriuretic peptides. In addition to cleaving membrane-bound proteoglycans and glycoproteins (most notably syndecan-1 and hyaluronic acid) off endothelial glycocalyx, they also reduce lymphatic drainage by inhibiting lymphatic propulsive motor activity (3, 4). This worsens FO because interstitial fluid is primarily channeled back into circulation via lymphatics.

Of note, some data regarding glycocalyx and natriuretic peptides is partly theoretical, and partly derived from adult studies and there is no pediatric-specific data in this context (8–10).

## Quantifying fluid overload

Despite the burgeoning evidence regarding association between FO and adverse patient outcomes, there is no consensus on the optimal method for calculating FO. Lombel et al. highlighted that depending upon the definition used, there was a significant variability in the number of patients identified with >10% FO (11).

Most pediatric studies utilize the FB method introduced by Goldstein et al. (12). The formula is:

$$\begin{array}{l} \%FO=\frac{\left(daily\;fluid\;intake\;\left(L\right)-total\;output\;(L)\right)}{Baseline\;body\;weight\;\left(kg\right)}x100 \end{array}$$

More practical WB(weight based) methods can be alternatively used to estimate degree of FO. The formulas are:

$$\begin{array}{l} \%FO=\frac{\left(\begin{array}{c} CRRT\;initiation\;weight\;\left(kg\right)\;-\\ \;hosptial\;admission\;weight\;\left(kg\right) \end{array}\right)}{hosptial\;admission\;weight\;\left(kg\right)}x100 \end{array}$$

or

$$\begin{array}{l} \%FO=\frac{\left(\begin{array}{c} CRRT\;initiation\;weight\;\left(kg\right)-\\ \;\;ICU\;admission\;weight\;\left(kg\right) \end{array}\right)}{ICU\;admission\;weight\;\left(kg\right)}x\;100. \end{array}$$

These are particularly useful in neonates because of uncertainties in estimating fluid input via breastmilk, fluid lost into diapers/bedsheets, and insensible losses apart from losses which can be markedly influenced by a number of factors (13). ICU admission weights when taken as baseline might under-estimate degree of FO in comparison to hospital admission weight. This discrepancy may also delay CRRT (continuous renal replacement therapy) initiation as most clinicians still consider 10% as the threshold to intervene (14). Multiple studies in both adult and pediatric literature have demonstrated poor agreement between changes in body weight and FO (**Table 2**). Nonetheless, reports have shown correlation between the two and a similar predictive potential of mortality (15). These inconsistent results warrant further research to recognize the most appropriate method of FO measurement.

## Is fluid overload ever independent of AKI?

**Figure 2:**
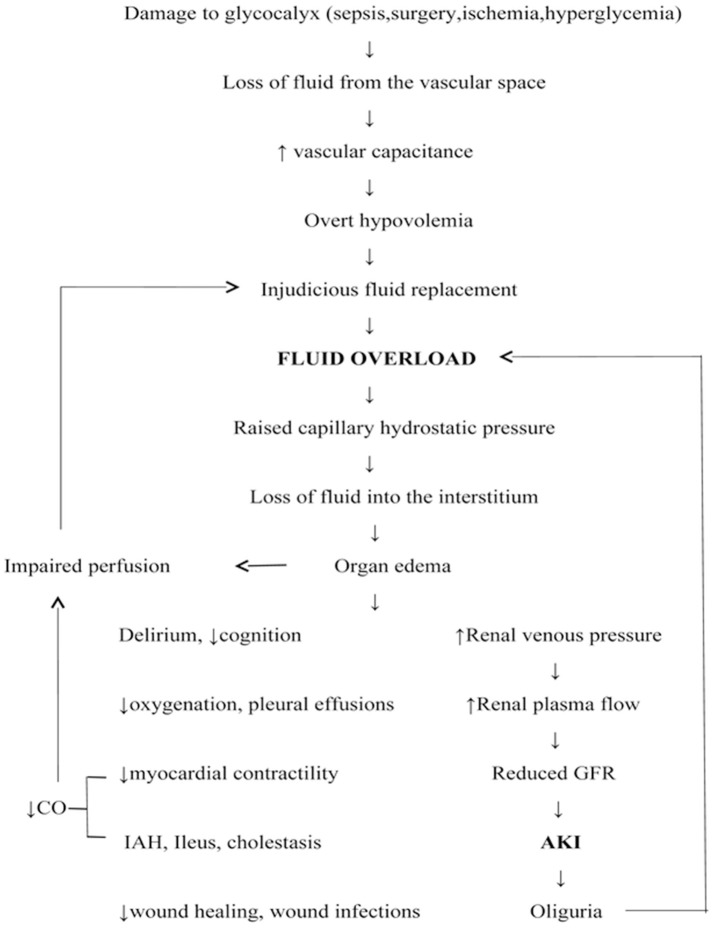
Fluid overload: pathogenesis and detrimental effects. CO, Cardiac output; AKI, acute kidney injury; IAH, Intra-abdominal hypertension; GFR, Glomerular filtration rate.

FO has detrimental effects on all organs, notably kidneys (2) (Figure 2). It is not just an epiphenomenon in AKI because FO *per se* renders an individual vulnerable to AKI and often predates it (23, 24). The development of abdominal hypertension and abdominal compartment syndrome impairs renal perfusion, and can culminate in a vicious cycle of AKI and FO (25). Deferral of AKI diagnosis correlates with the degree of fluid overload (Table 1). The risk of FO in this setting is that it can increase the volume of distribution of serum creatinine, a measure of AKI, leading to an overestimation of kidney function and a delayed diagnosis of AKI (26, 27). In scenarios where FO was not accounted for, patients with a decreasing trend in sCr (serum creatinine) had increased mortality than patients with high sCr and severe AKI (28).

**Table 1:** Common methods to calculate the degree of fluid overload.

**Fluid balance based methods**	**Weight based methods**
1. $\frac{Fluid\;in-\;fluid\;out}{PICU\;adm\;wt}\;x\;100$	1. $\frac{Daily\;wt-\;PICU\;adm\;wt}{hosp\;adm\;wt}\;x\;100$
2. $\frac{Fluid\;in-\;fluid\;out}{hosp\;adm\;wt}\;x\;100$	2. $\frac{Daily\;wt-hosp\;adm\;wt}{hosp\;adm\;wt}\;x\;100$
3. $\frac{Fluid\;in-\;fluid\;out}{PICU\;dry\;wt}\;x\;100$	3. $\frac{Daily\;wt-\;PICU\;dry\;wt}{PICU\;dry\;wt}\;x\;100$

*adm, admission; wt, weight; hosp, hospital*.

The following equation can be used to adjust sCr for fluid accumulation: adjusted Cr = sCr [1+ (Net fluid balance/Total body water)], where TBW = 0.6 × weight (kg) (29). However, use of the constant 0.6 in estimating TBW may not be accurate in all patients as it can be affected by age, sex, and various pathologic states (heart failure, cirrhosis, nephrosis).

Re-analysis of FACTT (Fluid and Catheter Treatment Trial) showed that presence of covert AKI was independently associated with a higher risk of death. Incidence of AKI was greater in the conservative arm before adjustment but in liberal arm post-adjustment (30). Secondary analysis of PICARD (Program to Improve Care in Renal Disease) cohort also revealed that correction for %FO uncovered missed diagnoses and reclassified AKI in some patients. Although no mortality differences were noted, the need for subsequent dialysis was higher in the misdiagnosed patients (27). Basu et al. reiterated these findings in 92 infants undergoing arterial switch operation wherein correction of sCr for FO not only increased AKI prevalence but also strengthened its association with postoperative morbidities (29).

## Epidemiology and outcomes

### Fluid overload and adverse outcomes: does current evidence endorse causality?

With the exception of select adult trials and recent findings of Grams et al. (31–36), no pediatric study to date has demonstrated FO as a causal contributor to mortality rather than just a marker of disease severity. In their *post-hoc* analysis of FACTT, Grams et al. showed FO as a causal intermediate in the association between diuretic administration and decreased mortality in AKI patients, suggesting that the mortality benefit associated with furosemide was due to a decrease in FO (35). Few pediatric studies have in fact shown that FO is a significant co-morbidity that is highly associated with adverse outcomes only in patients with limited disease unlike patients with severe disease in whom the ultimate outcome is independent of %FO, suggesting that the disease severity itself is the primary determinant (37–39).

### Renal replacement therapy

There are many indications for initiating renal replacement therapy (RRT) in a critically ill patient, FO being one of them. RRT provides control in fluid management and allows for the ability to achieve a negative fluid balance (40). Association between FO and mortality was first observed by pediatricians in critically ill children with AKI requiring RRT. Several pediatric studies have demonstrated an independent association between FO at the time of continuous renal replacement therapy (CRRT) initiation with mortality (41–45). A study by Goldstein looked at 116 pediatric intensive care unit patients with multiorgan dysfunction who received CRRT. The results showed a significant survival different in patients who were in a state of FO while on CRRT vs. those who are able to achieve their dry weight (36 vs. 76%) (43). The results from the ppCRRT Registry stratifying 297 patients into <10%, 10–20%, and >20% FO groups remain most comprehensive in this regard (41). Analysis revealed that presence of FO at CRRT initiation was associated with raised odds for mortality (aOR 1.03) after controlling for the severity of illness, with the maximum risk noted in the cohort with >20% FO(aOR 8.5,95% CI 2.8–25.7). The RENAL study (Randomized Evaluation of Normal vs. Augmented Levels of renal replacement therapy) showed that a negative mean daily FB was significantly associated with reduced mortality (*p* < 0.0001) and increased RRT-free days (*p* = 0.0017) (46). Non- surviving CRRT recipients (*n* = 610) in the PICARD study also had a significantly higher %FO at CRRT initiation (*p* = 0.01) and cessation (*p* = 0.004) (47). A subsequent re-analysis highlighted for the first time an increased risk of sepsis in patients after AKI. Authors speculated that FO compromises epithelial integrity by causing gut edema, thus favoring bacterial translocation (48).

### Extracorporeal membrane oxygenation

Patients on ECMO are vulnerable to develop significant degree of FO due to the inflammatory milieu triggered by their critical illness, exposure to extracorporeal circuit and iatrogenic administration of fluids to minimize venous access insufficiency. The association between FO and mortality in children on ECMO has been reaffirmed in various pediatric and adult cohorts. Per literature, the need for initiating RRT arises in almost 50% ECMO patients (49, 50). Selewski et al. recently showed that median FO at CRRT initiation and discontinuation were significantly lower in ECMO survivors (49). FO reduction to <10% at CRRT conclusion was not associated with improved survival, emphasizing that early intervention is the key to enhanced survival. Although a growing body of evidence favors concomitant use of CRRT in ECMO patients, reports on this issue have been contentious (51, 52). In another study, Selewski showed that the degree of peak FO was associated with significant increases in duration of ECMO. Patients who died during hospitalization had higher FO at ECMO initiation, along with higher peak FO. Also noted was a graded increase in both ECMO and hospital mortality by 10% interval increases in FO (**Table 4**) (40).

### Cardiac surgery

In neonates with congenital heart disease undergoing cardiac surgery, fluid management is of particular importance. These patients are administered large amounts of fluid pre, intra, and post-operatively to prevent hypotension. The mainstay of management in these patients is diuretic therapy, with furosemide being the most widely administered drug (16). FO in these patients can also result from AKI (incidence = 30–50%) and systemic inflammation stemming from cardiopulmonary bypass (CPB) (53). FO in this cohort is significantly associated with increased in-hospital mortality (15, 54, 55); longer ICU and hospital stay, prolonged inotrope and ventilator support, and delayed sternal closure (56–59). Novel FO mitigating strategies, namely passive peritoneal drainage and modified ultrafiltration techniques are increasingly being employed. Given the high risk status of CHD patients, many are considered for preventative use of PD to reduce fluid accumulation. In some institutions the practice of inserting PD catheters intra-operatively is considered routine (16). Several studies have purported that prophylactic PD (peritoneal dialysis) achieves a net negative FB, ameliorates post-CPB inflammation via cytokine removal and reduces inotrope requirement (60–62). However, few single center studies do not reaffirm above findings (63). Most recent data on this population suggests that the potentially deleterious FO cut-off is likely <10% as studies have demonstrated equally poor outcomes with a FO level as low as 5% (54, 59).

### Neonates

Association between FO and BPD (bronchopulmonary dysplasia) has been an active area of research. Studies document an association between FB and weight loss in the first few weeks of life and subsequent development of BPD in preterm neonates (64–66). In the neonatal period, a physiologic contraction of the extracellular fluid compartment leads to post-natal weight loss and a negative fluid and sodium balance. When this process is hindered by excess fluid intake, the retention of extracellular fluid can lead to fluid accumulation in pulmonary interstitial tissue. This puts the neonate at risk of barotrauma leading to BPD (64). Askenazi et al. reported that neonates with AKI had a higher maximum weight change in first 4 days of life (*p* = 0.05), higher risk of death and were more likely to require prolonged MV (*p* < 0.03) and oxygen support than neonates without AKI even post-adjustment (67). These studies suggest that ameliorating FO and AKI can reduce mortality and their sequelae (BPD and chronic kidney disease) which incur long term morbidity and excessive medical costs (Tables 3, 4). In the treatment of BPD, diuretics have been used; though studies have only shown short term effects without any long term benefits (80). Literature has suggested the possible benefit of practicing fluid restriction in preterm infants to prevent BPD. However, recent systematic review showed no evidence that supported fluid restriction in infants would prevent the subsequent development of BPD (81).

**Table 2:** Studies assessing the correlation and agreement between the fluid balance and weight based methods of fluid overload estimation.

**Study author**	**Conclusion**
van Asperen et al. (13)	Fluid balance charts both over and under-estimate body weight change and are unreliable as a single measure of fluid status in neonates
Selewski et al. (15)	Both the methods were similar in predicting the degree of FO at CRRT initiation and mortality
Hazle et al. (16)	Both the methods could predict the significant association between positive fluid balance and associated poor outcomes in infants post-cardiac surgery
Benoit et al. (17)	>10% weight gain (*p* = 0.001) and fluid overload (0.075) predicted PICU admission in pediatric stem cell transplant recipients on univariate analysis but only >10% weight gain (0.018) remained an independent risk factor for PICU admission after adjustment
Bontant et al. (18)	- Correlations were strong between fluid input minus output/adjusted fluid input minus output and body weight change - Agreement between was poor between fluid input minus output/adjusted fluid input minus output and body weight change during the first 24 h after PICU admission - Since daily body weight is not particularly difficult to measure, fluid input minus output/adjusted fluid input minus output calculations may be reserved for the most severely ill patients in whom body weight measurement is strictly contraindicated
Perren et al. (19)	Correlation and Bland Altman agreement was poor between - body weight change and cumulative fluid balance in a cohort of ICU patients
Mank et al. (20)	- Body weight was deemed to be a more accurate, safe and reliable parameter for monitoring fluid retention in patients undergoing hyperhydration prior to chemotherapy - Correlation between body weight change and cumulative fluid balance was relatively low
Eastwood (21)	Body weight gain in post-cardiac surgery patients was falsely undermined by the fluid balance method
Kelm et al. (22)	Fluid balance did not correlate with clinical and radiological evidence of FO in a large cohort of septic patients but body weight did

FB method: $\%FO=\frac{\left(dailyfluidintake\left(L\right)-totaloutput\left(L\right)\right)}{Baselinebodyweight\left(kg\right)}x\text{100}.$

Weight based methods: $\%FO=\frac{\left(CRRTinitiationweight\left(kg\right)-hosptialadmissionweight\left(kg\right)\right)}{hosptialadmissionweight\left(kg\right)}x\text{100}.$

Or

$\%FO=\frac{\left(CRRTinitiationweight\left(kg\right)-ICUadmissionweight\left(kg\right)\right)}{ICUadmissionweight\left(kg\right)}x\text{100}.$

**Table 3:** Impact of FO on various outcome variables in the given pediatric populations.

**Author; Year**	**Outcome measures considered**	**Observations**
**RENAL REPLACEMENT THERAPY**		
Sutherland et al. (41)	Mortality	FO at CRRT initiation with raised odds [aOR 1.03] for mortality [≥20, 10–20, <10% FO groups with 65, 43, and 29% mortality respectively].
Hayes et al. (42)	Mortality	Median FO at CRRT initiation was 7.3% in survivors vs. 22.3% in non-survivors.
Goldstein et al. (43)	Mortality	Mean FO at CRRT initiation was 25.4% in survivors vs. 14.2% in non-survivors.
Gillespie et al. (44)	Mortality	≥10%FO at CRRT initiation significantly increased the risk of mortality [HR 3.02, 95%CI 1.5–6.1; *p* = 0.002].
Foland et al. (45)	Mortality	Median FO was significantly lower in survivors vs. non-survivors [7.8 vs. 15.5%; *p* = 0.01].
Goldstein et al. (12)	Mortality	Mean FO was significantly lower in survivors [16.4%] than non-survivors [34%] after controlling for illness severity [*p* = 0.03].
**EXTRACORPOREAL MEMBRANE OXYGENATION**		
Selewski et al. (49)	Mortality	Median FO at CRRT initiation significantly lower in survivors compared to non-survivors [24.5 vs. 38%, *p* = 0.006]. FO<10% at CRRT initiation was associated with reduced odds of mortality [aOR 0.02, 95%CI 0.00–0.07, *p* = 0.035].
Swaniker et al. (68)	Mortality	Mean BW decreased about 5 ± 2% in survivors vs. increasing 11 ± 5% [*p* < 0.001] in non-survivors.
Selewski et al. (40)	Mortality	Increased ECMO mortality was associated with >20% FO at ECMO initiation (35.2 vs. 22%; *p* = 0.0003). During ECMO, a peak FO >30% was associated with increased ECMO mortality (34 vs. 15.9%; *p* < 0.0001).
**CARDIAC SURGERY**		
Lex et al. (54)	Mortality, LCOS, LMV	>5%FO was independently associated with mortality [aOR, 1.14,95%CI 1.008–1.303; *p* = 0.041] and LCOS [1.21,95% CI 1.12–1.30, *p* = 0.001].
Sampaio et al. (56)	LMV, LOS	Maximum cumulative fluid balance [6.82 %(IQR 3.28–11.71)] was associated with duration of MV [adjusted β coefficient = 0.53, CI 0.38–0.66, *p* < 0.001], LOS in PICU [Spearman's ρ = 0.45, *p* < 0.001].
Piggottv et al. (57)	LOS, LMV, mortality	>15%FO was associated with higher mortality [31% vs. 0%; *p* < 0.001], greater median LOS and LMV [39 vs. 76 days, *p* = 0.03 and 8 vs. 25 days, *p* < 0.001 respectively].
Seguin et al. (58)	LOS, LMV, OI	Peak cumulative FO during day 2 predicted longer LOS [aHR 0.95, 95%CI 0.92–0.99, *p* = 0.009], longer MV [aHR 0.97, 95%CI 0.94–0.99, *p* = 0.03].
Hassinger et al. (59)	LOS, LMV, inotropic support, AKI	≥5%FO was independently associated with prolonged need for MV, LOS, inotropic support [*p* < 0.001] and post CPB-AKI [*p* = 0.023].
Hazlev et al. (16)	LOS, LMV, mortality	Higher mean max FO by both FB [12 ± 10 vs. 6 ± 4%, *p* = 0.03] and weight based [24 ± 15 vs. 14 ± 8%, *p* = 0.02] methods was associated with composite poor outcome.
Basu et al. (29)	AKI	Infants who developed AKI after surgery had higher fluid balance [148 ± 125 vs. 115 ± 117ml/d, *p* = 0.016].
Grist et al. (55)	Mortality, LOS	Mean positive balance in non-survivors was 18ml/kg. FO was associated with increased mortality [aOR1.73 (95% CI 1.01–2.96)] and longer LOS [*p* < 0.05].
**NEONATES**		
Lee and Cho (69)	Mortality	Neonates with ≥30% FO at the time of CRRT initiation had lower survival rates [*p* = 0.009].
Askenazi et al. (67)	AKI	Median weight gain at D3 of life was higher in the AKI vs. non AKI cohort [8.2% IQR (4.4–21.6%) vs. −4% (IQR−6.5 to 0.0%) (*p* < 0.001)]. Infants with AKI had lower survival rates than those without AKI [72 vs. 100 % (*p* < 0.02)].
Askenazi et al. (70)	MV/death, AKI	Infants with AKI [30%] had a higher max% weight change in the first 4 days of life [*p* = 0.05] and were at higher risk of death/MV at D28 [*p* < 0.03].
**RESPIRATORY TRACT DISEASE**		
Ingelse et al. (71)	LMV	Higher D3 CFB was independently associated with prolonged LMV [β = 0.166, *p* = 0.048].
Sinitsky et al. (72)	OI, LMV	FO% had significant correlation with OI [Spearman ρ 0.318; *p* < 0.0001] and invasive ventilation days [ ρ 0.274; *p* < 0.0001].
Willson et al. (32)	Mortality, VFDs, OI	Mean CFB in non-survivors was significantly higher than survivors [8.7 ± 9.5L/m^2^ vs. 1.2 ± 2.4L/m^2^; *p* < 0.001]. Higher CFB was significantly associated with fewer VFDs (*p* < 0.001) and higher OI [0.52 point increase in OSI for each L/m^2^ increase in FB; *p* = 0.011].
Valentine et al. (73)	VFDs	Higher CFB at D3 was independently associated with fewer VFDs [*p* = 0.02].
Arikan et al. (33)	LMV, PICU and hospital LOS	≥15%FO were both independently associated with LMV [*p* = 0.004 and 0.01], PICU stay [*p* = 0.008 and 0.01] and hospital LOS [*p* = 0.02 and 0.04]. Peak total FO, OI and PELOD score in survivors and non-survivors were 13.7 ± 10.0 vs. 15.9 ± 10.3 (*p* = 0.45); 16.6 ± 17.6 vs. 31.5 ± 29.6 (*p* = 0.05) and 18.2 ± 7.6 vs. 20.9 ± 9.5 (*p* = 0.01) respectively.
Floriv et al. (74)	Mortality, VFDs	Positive FB (in increments of 10 mL/kg/24 h) was significantly associated with increased mortality [OR1.08, 95% CI 1.01–1.15, *p* = 0.02] and reduced VFDs [−0.21 (−0.39 to −0.04), *p* = 0.02].
**SEPSIS**		
Abulebda et al. (37)	Mortality	Median CFB in non-survivors was 19.5% vs. 6.5% in survivors [*p* < 0.001].
Chen et al. (75)	Mortality	Both early FO and PICU-acquired daily FO of >5% were associated with mortality [aOR 1.20; 95%CI 1.08–1.33; *p* = 0.001; *n* = 202 and aOR = 5.47 per log increase; 95%CI 1.15-25.96; *p* = 0.032; *n* = 154]. Median values of early and acquired daily FO in survivors vs. non-survivors were 0.62% [−0.47 to 2.19] vs. 3.00% [−0.23 to 5.28] and 0.27% [1.66 to 4.61] vs. 4.27% [2.74 to 6.56] respectively.
**MISCELLANEOUS**		
Li et al. (34)	Mortality, AKI	Early FO (>5% in first 24h) was independently associated with AKI (OR 1.34, *p* < 0.001) and mortality (OR 1.36, *p* < 0.001). Incidence of AKI and mortality in children with early FO was 18.8 vs. 4.2 and 15.6% vs. 2.6% respectively when compared to children with < 5%FO.
Bhaskar et al. (76)	Mortality	Early FO (>10% in 72h) [aOR 9.17, 95 %CI 2.22–55.57], its severity [aOR 1.11,1.05–1.19] and duration [aOR 1.61, 1.21–2.28] independently predicted mortality. Cases had significantly higher mortality than controls [26 vs. 6%; *p* = 0.003], even in the matched analysis [37 vs. 3 %; *p* = 0.002].

*LOS-length of stay; LMV-length of mechanical ventilation; LCOS-low cardiac output syndrome; VFD-ventilator free days; aOR-adjusted odd ratio; HR-hazard ratio*.

**Table 4:** Summary of the studies evaluating outcomes associated with fluid overload in different pediatric populations.

**Author; Year**	**Study design**	**Study sample [*n*; age group; inclusion criteria]**	**Substantive evidence (regarding association of)**	**Relevant findings**
**RENAL REPLACEMENT THERAPY**				
Goldstein et al. (12)	Retrospective observational Single center 1996–1998	21 children 8.8 ± 6.3 y PICU patients receiving CVVH±D	%FO at CVVH/D initiation with poor outcomes in critically ill children	- FO% was significantly lower in survivors (16.4 ± 13.8%) than nonsurvivors (34 ± 21%) after controlling for illness severity (*p* = 0.03)
Foland et al. (45)	Retrospective observational Single center 1997–2003	113 children 9.6 y (2.5–14.3) ICU children receiving CVVH	%FO prior to CVVH and mortality	- Median FO% was significantly lower in survivors vs. nonsurvivors [7.8 vs. 15.5%; *p* = 0.01] - FO% at CVVH initiation was independently associated with mortality in patients with ≥ 3 organ MODS (*p* = 0.01)
Gillespie et al. (44)	Retrospective observational Single center 1993–2002	77children ≤20y Patients on CVVH	%FO at the time of CVVH initiation with mortality	- High FO (≥10%) at CRRT initiation significantly increased the risk of mortality [HR 3.02, 95%CI 1.5–6.1; *p* = 0.002]
Goldstein et al. (43)	Prospective observational (ppCRRT) Multicenter	116 children <18 y MODS patients on CRRT	%FO prior to CRRT initiation with mortality in MODS patients	%FO at CRRT initiation was significantly lower for survivors than non-survivors even after adjusting for severity of illness Similar findings for patients receiving mechanical ventilation and vasoactive pressors, even after adjusting for illness severity (*p* < 0.05)
Hayes et al. (42)	Retrospective observational Single center 2000–2005	76 children 5.8 y (0–19) PICU patients with AKI needing CRRT	% FO at CRRT initiation with mortality	- Median %FO at CRRT initiation was 7.3% in survivors vs. 22.3% in nonsurvivors (*p* = 0.0001) - ≥20% FO at CRRT initiation was significantly associated with mortality (*p* = 0.006), LMV (*p* = 0.018), PICU stay (*p* = 0.0425), hospital LOS (*p* = 0.0123)
Sutherland et al. (41)	Prospective observational (ppCRRT) Multicenter 2001–2005	297 children <18 y ICU children on CRRT	%FO with mortality in children receiving CRRT	- FO of ≥20% was independently associated with increased mortality (aOR 8.5, 95% CI 2.8–25.7) - Presence of FO at CRRT initiation was associated with raised odds [aOR 1.03] for mortality which was significantly higher for worse degrees of FO [≥20, 10–20, <10% FO groups with 65, 43 and 29% mortality respectively]
Selewski et al. (49)	Retrospective observational Single center 2006–2010	113 children 19 m (0.2–181) PICU patients on CRRT	Different weight based FO definitions with PICU mortality	- FO% at CRRT initiation was significantly greater in non-survivors via both weight based and FB method - Univariate OR for PICU mortality was 1.056 [95%CI 1.025–1.087] by fluid balance method, 1.044 [95% CI 1.019–1.069] by the PICU admission weight-based method and 1.045 [95% CI 1.022–1.07] by hospital admission weight-based method - On multivariate analyses, all three methods significantly predicted PICU survival
de Galasso et al. (38)	Retrospective observational Single center 2000–2012	131 children 0–18 y PICU patients on CRRT	FO with mortality only in children with milder disease	- FO>10% at CRRT initiation was associated with mortality only in children with milder disease [OR 10.9,95 %CI 0.78–152.62; *p* = 0.07]
**EXTRACORPOREAL MEMBRANE OXYGENATION**				
Hoover et al. (77)	Retrospective observational (Matched case control) 1992–2006 Single center	86 children 1 m−18 y Patients on ECMO vs. those on ECMO+CVVH	Improved fluid balance, caloric intake and less furosemide use with ECMO+CVVH cf ECMO alone	In ECMO survivors who received CVVH, median FB was less than that in non-CVVH survivors [25.1 vs. 40.2ml/kg/d; *p* = 0.028] - Use of CVVH was associated with earlier optimal caloric intake (*p* < 0.001) and reduced diuretic administration (*p* = 0.009) - Survival did not differ significantly in the two groups (*p* = 0.51)
Blijdorp et al. (78)	Retrospective observational (1:3 matched case- comparison) 2002–2006 Single center	61 neonates <28 d Patients on ECMO vs. those on ECMO+CVVH	Better fluid balance via HF in ECMO patients with improved outcomes	- Median time on ECMO [98 h,IQR 48–187 vs. 126 h,24–403; *p* = 0.02] and MV post decannulation [2.5d vs. 4.8d; *p* = 0.04] was significantly shorter in HF group
				- Cost per ECMO run and blood transfusion requirement was also reduced (*p* < 0.001) - No mortality benefit was noted in the HF group
Paden (52)	Retrospective observational 1997–2007 Single center	68 children <19 y Patients on ECMO+CRRT	Recovery of renal function and survival with HF during ECMO	- In the absence of primary renal disease, chronic renal failure did not occur following concurrent use of CRRT with ECMO - Mortality is higher in patients receiving concomitant CRRT and ECMO compared to those receiving ECMO, but is similar to patients requiring CRRT who are not on ECMO
Selewski et al. (40)	Retrospective cohort Multicenter 2007–2011	756 children <18 y Patients on ECMO	Survival associated with peak FO during ECMO and %FO at ECMO initiation	- A significantly higher hospital mortality risk was associated with each 10% rise of FO at initiation of ECMO (OR, 1.12; 95% CI, 1.04–1.19 *p* = 0.002). - With each 10% rise in peak FO during ECMO, there was a 9% increased odds of ECMO mortality and a 17% increased odds of hospital mortality
**CARDIAC SURGERY**				
Grist et al. (55)	Retrospective observational Single center 2003–2009	1570 children Underwent congenital heart surgery with CPB and MUF	FO with mortality in children undergoing CPB	- 20% (314) children had a positive FB - Patients with positive FB weighed more, had higher RACHS score, longer pump time, longer LOS with an aOR for mortality of 1.73 (95% CI 1.01–2.96)[*p* < 0.05 for all variables]
Saini et al. (60)	Retrospective observational (1:1 matched case control) Single center 2006–2010	36 infants <1 y Post- atrioventricular septal defect repair ±PD insertion	Improved fluid balance with passive PD insertion post AVSD repair in infants	Infants with passive PD achieved negative fluid balance more rapidly (12 ± 10 vs. 27.3 ± 13 h, *p* < 0.0001) and to a greater extent (*p* = 0.002)
Hazle et al. (16)	Prospective observational Single center 2009–2010	49 infants <6 m Underwent congenital heart surgery	Postop FO with longer LOS, MV and mortality in infants undergoing cardiac surgery	- Higher mean max FO by both FB (12 ± 10 vs. 6 ± 4%, *p* = 0.03) and weight based (24 ± 15 vs. 14 ± 8%, *p* = 0.02) methods was associated with composite poor outcome - FO < 10% was associated with a good outcome by both FB and weight based methods (*p* = 0.02,0.01) but the association did not reach statistical significance after multivariate analysis
Sasser et al. (61)	Prospective before and after nonrandomized cohort Single center 2010–2011	52 neonates and infants Underwent congenital cardiac surgery with (25) or without (27) prophylactic PD use	Greater net negative FB with prophylactic PD placement post-CBP in infants	- Median net fluid balance was more negative in +PD at 24 and 48 h [−24 mL/kg (IQR-62,11) vs. +18 (−26, 11), *p* = 0.003; - 88 (−132,−54) vs.-46 (−84,–12), *p* = 0.004] - Duration of MV (*p* = 0.1), mean inotrope score (*p* = 0.04) and serum IL-6 and 8 levels were lower in +PD at 24h - Median time to sternal closure was lower in +PD [24 h (IQR 20–40) vs. 63 (44–72), *p* < 0.001]
Basu et al. (29)	Retrospective observational Single center 1997–2008	92 children 5.5 days (4–7.5) Status post arterial switch operation	Delayed AKI diagnosis with unadjusted sCr (not accounting for positive FB)	- Infants who developed AKI after surgery had higher POD1 FB [148 ± 125 vs. 115 ± 117ml/d, *p* = 0.016] - Correcting sCr for FO increased AKI prevalence and strengthened its association with postop morbidities
Seguin et al. (58)	Retrospective observational Single center 2005–2007	193 patients <18 y Post cardiac surgery	Early FO with LOS, MV and OI	- Early postop fluid administration was independently associated with higher D2 FO% (*p* = 0.0001) - D2 FO% predicted longer LOS (aHR 0.95, 95%CI 0.92–0.99, *p* = 0.009), longer MV (aHR 0.97, 95%CI 0.94–0.99, *p* = 0.03) - Higher daily FO% predicted worse daily OI in patients without cyanotic heart disease (aHR 0.16, 95%CI 0.07–0.25, *p* = 0.03)
Hassinger et al. (59)	Secondary analysis of prospective observational study Single center	98 children 2 wks−18 y Status post-CPB	Early postop FO with prolonged LOS, LMV, inotropic support and AKI development	- Early postop FO (≥5%) was independently associated with prolonged need for MV, LOS and inotropic support (*p* < 0.001) - FO was associated with post-CPB AKI; FO more often preceded than followed it but AKI was not consisitently associated with FO - Cumulative fluid administered was an excellent predictor of modified pRIFLE category [AUC = 0.96, 95%CI 0.92–1, −*p* = 0.002] - Patients with FO were administered higher fluid volume (*p* < 0.001) and had a poor urinary response to diuretics
Kwiatkowski et al. (62)	Retrospective observational (1:1 matched case control) 2007–2012	84 infants <6 m Underwent congenital heart surgery ± PDC insertion	Improved FB via elective PDC use with favorable outcomes in infants undergoing cardiac surgery	- PDC+ group had higher negative fluid balance on POD 1 and 2 (57 vs. 33%, *p* < 0.04; 85 vs. 61%, *p* < 0.01) - PDC+ group had shorter time to negative FB (16 vs. 32 h, *p* < 0.0001), earlier extubation (80 vs. 104h, *p* < 0.02), improved inotrope scores (*p* < 0.04), and fewer electrolyte imbalances requiring correction (*p* < 0.03)
Piggott et al. (57)	Retrospective observational Single center 2010–2013	95 neonates 6–29 d Underwent congenital cardiac surgery	Postop FO with prolonged MV, LOS and mortality	- AKI in 45% neonates - >15%FO was associated with prolonged LOS (*p* = 0.03), postop ventilator days (*p* < 0.001) and mortality (*p* < 0.001) - Certain risk factors like preop aminoglycoside use, selective cerebral perfusion, CPB time, small kidneys on US can be modified to minimize risk of AKI and perhaps FO - Prophylactic PD catheters can be placed in infants with small kidneys identified preoperatively to avert FO
Sampaio et al. (56)	Retrospective observational 2010–2013	85 children <17 y Surgery for congenital heart disease+ MV for at least 12h in PICU	FO with prolonged MV and LOS in patients post-congenital heart surgery	- Maximum CFB was associated with duration of MV (adjusted β coefficient = 0.53, CI 0.38–0.66, *P* < 0.001), LOS in PICU (Spearman's ρ = 0.45, *P* < 0.001), and presence of chest wall edema and pleural effusions on chest radiograph (*p* = 0.003)
Lex et al. (54)	Secondary analysis of a prospective observational study Single center 2004–2008	1,520 children <18 y Underwent open heart surgery	Early postop FO with higher mortality and morbidity	Higher FO on the day of surgery was independently associated with mortality (aOR, 1.14,95%CI 1.008–1.303; *p* = 0.041) and LCOS (1.21,95% CI 1.12–1.30, *p* = 0.001) - Higher maximum s.Cr (aOR 1.01,1.003–1.021; *p* = 0.009), maximum vasoactive-inotropic score (aOR 1.01,95% CI 0.005–1.029; *p* = 0.042) and higher blood loss on the day of the surgery (aOR1.01, 95%CI 1.004–1.025; *p* = 0.015) were associated with a higher risk of >5%FO
**NEONATES**				
Ohv et al. (64)	Secondary analysis of the RCT by the Neonatal Research Network Multicenter 1999–2001	1,382 neonates ELBW newborns with birth wt between 401 and 1,000 g	Positive FB in the first 10 days of life with death/BPD	- 58% either died or developed BPD; 42% survived without BPD - Higher fluid intake (*p* < 0.001) or lower weight loss (*p* = 0.06) during first 10 d were significantly associated with death/BPD - Lower BW/GA/1.5 min Apgar scores, higher O_2_ requirement at 24 h of life and longer LMV were associated with death/BPD
Schmidt et al. (65)	Secondary analysis of TIPP (Randomized controlled trial of Indomethacin prophylaxis in preterms) Multicenter	999 neonates Extremely low birth weight newborns who survived to a postmenstrual age of 36 wks	- Positive FB in preemies with subsequent BPD - Uncertainty about cause-and-effect relationship between PDA and BPD	- Neonates without PDA who received prophylactic indomethacin had lower urinary output, lower weight loss (*p* = 0.012) and higher FiO_2_ requirement (*p* < 0.0001) by the end of first week - Incidence of BPD was similar in PDA+ neonates irrespective of indomethacin prophylaxis but was significantly higher in PDA- infants who received indomethacin (*p* = 0.015) - Indomethacin prophylaxis reduces the incidence of PDA but not that of BPD
Askenazi et al. (70)	Prospective observational Single center 2010–2011	58 neonates near term (≥34 wks and >2,000g) and term with Apgar score ≤7	AKI with FO and mortality in sick near term/term neonates	- AKI in 15.6% neonates - Median weight gain at D3 of life was higher in the AKI vs. non AKI cohort [8.2%, IQR 4.4–21.6% vs. −4%, IQR −6.5 to 0.0% (*p* < 0.001)] - Infants with AKI had lower survival rates than those without AKI [72 % vs. 100 % (*p* < 0.02)]
Askenazi et al. (67)	Prospective observational Single center 2012–2013	122 preterm neonates <31wks, <1,200 g	- FO with prolonged need for oxygen support, MV and mortality - AKI with BPD and mortality	- Infants with AKI (30%) had a higher max% wt change in the first 4 days of life (*p* = 0.05)and were at higher risk of death/MV at D28 (*p* < 0.03) - Although infants with FO had an increased RR to receive oxygen support/death (1.02, 95%CI 1.01–1.03; *p* < 0.0001) and MV/death (1.03,1.02–1.05; *p* < 0.0001) at D28, after adjustment this trend did not reach statistical significance (*p* = 0.16,0.06) - Similar finding was noted for time taken to oxygen weaning [HR 0.97 (0.9–0.99), *p* < 0.02; aHR 0.98 (0.9–1.01), *p* = 0.18]
Lee and Cho (69)	Retrospective observational Single center 200–2014	34 neonates (15 preterm, 19 term) Admitted to NICU On CRRT for ≥24 h	Higher %FO at CRRT initiation with mortality	- Neonates with ≥30% FO at the time of CRRT initiation had lower survival rates - Univariate Cox regression analysis revealed that a higher %FO at CRRT initiation and decreased urine output at the end of CRRT were associated with mortality - Multivariate Cox regression analysis showed that decreased urine output at CRRT conclusion was associated with mortality
**RESPIRATORY TRACT DISEASE**				
Sinitsky et al. (72)	Retrospective observational Single center 2009–2013	636 children <16 y Mechanically ventilated PICU patients	Early FO with respiratory morbidity in PICU patients	- FO% had significant correlation with OI [Spearman ρ 0.318; *p* < 0.0001] and invasive ventilation days [ ρ 0.274; *p* < 0.0001] - FO% at 48 h was significant predictor of both OI (*p* < 0.001) and ventilation days (*p* = 0.002) - No association of FO% at 48 h with mortality
Flori et al. (74)	*Post hoc* analysis of a prospective observational study Multicenter 1996–2000	320 children <18 y Mechanically ventilated patients with ALI	FO with mortality and respiratory morbidity in children with ALI	- Positive FB (in increments of 10 mL/kg/24 h) was significantly associated with increased mortality [OR1.08, 95% CI 1.01–1.15, *p* = 0.02] and reduced VFDs [−0.21 (−0.39 to −0.04), *p* = 0.02], even after adjusting for multiple organ system failure, sepsis and the extent of oxygenation defect
Valentine et al. (73)	Retrospective observational Multicenter 2007–2010	168 children 1 m−18 y Mechanically ventilated patients with ALI	FO with fewer VFDs in children with ALI	- Higher CFB at D3 was independently associated with fewer VFDs (*p* = 0.02) - No association with mortality was noted (*p* = 0.11)
Ingelse et al. (71)	Retrospective observational Single center 2008–2014	135 children <2 y Mechanically ventilated PICU patients with viral lower respiratory tract disease	Early FO with prolonged LMV	- Mean CFB on D3 was 97.9 ± 49.2 mL/kg - Higher D3 CFB was independently associated with prolonged LMV [β = 0.166, *p* = 0.048] - No association found Between D3 CFB and sOSI (*p* = 0.7)
Willson et al. (32)	*Post hoc* analysis of the pediatric arm of an RCT Multicenter 2008–2010	110 children 0–18 y Mechanically ventilated children with ALI	FO with mortality, fewer VFDs and worse oxygenation	- Mean CFB in non-survivors was significantly higher than survivors [8.7 ± 9.5 vs. 1.2 ± 2.4L/m^2^; *p* < 0.001] - Higher CFB was significantly associated with fewer VFDs (*p* < 0.001) and higher OI [0.52 point increase in OSI for each L/m^2^ increase in FB; *p* = 0.011]
Arikan et al. (33)	Retrospective observational Single center 2004–2005	80 children 59 ± 73 months (mean ± *SD*) Mechanical ventilation for 24 h and presence of an indwelling arterial catheter	FO with prolonged LOS, LMV and impaired oxygenation	- Higher peak FO% predicted higher peak OI, independent of age, gender and PELOD scores (*p* < 0.009) - Peak FO% and severe FO% (≥15%) were both independently associated with prolonged LMV (*p* = 0.004 and 0.01), PICU stay (*p* = 0.008 and 0.01) and hospital LOS (*p* = 0.02 and 0.04)
**SEPSIS**				
Abulebda et al. (37)	Retrospective observational Multicenter	317 children <10 y Septic shock patients	FO with mortality only in low risk septic patients, barring the intermediate and high risk cohort	- Increased CFB was associated with mortality in the low risk cohort (*n* = 204,OR 1.035, 95%CI 1.004–1.066) but not in the intermediate and high risk cohorts - Higher FB in the first 24 h was not associated with mortality
Chen et al. (75)	Retrospective observational Single center 2011–2015	202 children 1 m−18 y Admitted to PICU with severe sepsis	Early and acquired daily FO with mortality in septic children	Both early FO (aOR 1.20; 95%CI 1.08–1.33; *p* = 0.001; *n* = 202) and PICU-acquired daily FO (aOR = 5.47 per log increase; 95%CI 1.15–25.96; *p* = 0.032; *n* = 154) were independent risk factors associated with mortality even after adjusting for illness severity - Median PICU LOS increased with greater fluctuations in FO [*p* < 0.001] - Early FO achieved an AUC of 0.74 (95% CI 0.65–l0.82; *p* = < 0.001; *n* = 202) for predicting mortality
**MISCELLANEOUS**				
Bhaskar et al. (76)	Retrospective observational (Matched case-control) Single center 2009–2010	114 children 0–17.4 y Admitted to PICU with shock	Early FO with mortality in shock patients	Early FO (>10% in 72h)[aOR 9.17, 95 %CI 2.22–55.57], its severity [aOR 1.11,1.05–1.19] and duration [aOR 1.61, 1.21–2.28] as independent predictors of mortality - Cases had significantly higher mortality than controls (26 vs. 6%; *p* = 0.003),even in the matched analysis (37 vs. 3 %; *p* = 0.002)
Liv et al. (34)	Prospective observational Single center 2011–2012	320 children 1 m−16 y Admitted to PICU for >24h	Early FO with AKI and mortality in critically ill children	- Early FO was independently associated with AKI (OR 1.34, *p* < 0.001) and mortality (OR 1.36, *p* < 0.001) - AUC of early FO for predicting mortality was 0.78 (*p* < 0.001)
Maitland et al. (79)	Open randomized controlled trial Multicenter 2009–2011	3,170 children 60 d−12 y Severe febrile illness with impaired perfusion Stratum A (3141)- saline/ albumin/no bolus Stratum B (29)– saline/albumin bolus in cases with severe hypotension	Fluid boluses with increased 48h mortality in critically ill children with impaired perfusion	- In stratum A, the 48 h mortality was 10.6,10.5,7.3% in the albumin-bolus, saline-bolus, and control group respectively - 28d mortality was 12.2, 12, and 8.7% in the three groups respectively (*p* = 0.004 for bolus vs. control) - In stratum B, mortality was 69% vs. 56% in the albumin vs. saline group respectively (*p* = 0.45)

### Acute respiratory distress syndrome

Several studies have demonstrated association between FO and worse respiratory outcomes (72–74). The adult FACTT trial showed that mean CFB (*p* < 0.001), VFDs (ventilator-free days) and ICU-free days were significantly lower in the conservative arm (31). Similarly, Valentine et al. noted that daily FO on D1-3 and CFB on D1-7 were higher in children with FO compared to adults in FACTT conservative arm (*p* < 0.001, each day) but similar to adults in the liberal arm, and promulgated the use of a Bayesian pediatric trial mirroring FACTT. No association with mortality was noted (73). A simplified fluid restrictive strategy employed in “FACTT lite” study also led to significantly lower FO than FACTT liberal protocol and no difference in VFDs compared to FACTT conservative protocol (82). Interestingly, mortality has been shown to correlate with increasing CFB (46), but not to a restrictive fluid management (31, 82).

## Use of early goal directed resuscitation- has the impasse ended?

The early goal directed therapy (EGDT) has been an area of intense research since its inception in the landmark Rivers' study wherein a mortality benefit was sought using protocolized (maintaining a CVP of 8–12 mmHg and SvO_2_ ≥70%) fluid resuscitation within first 6 h in the treatment of septic patients (83). An early up-front aggressive resuscitation was effective but late and excessive fluid administration was associated with worse survival at hospital discharge, 28 and 60 days. The critical difference emphasized by this protocol was the timing of the administration since the overall amount of fluid administered was equal in the two groups: a corollary to the conclusions drawn by Arikan et al., who recently evaluated outcomes in a controlled before and after study using a protocol driven resuscitation bundle in children with septic shock (84). Even though the total volume of fluid boluses did not differ between the two groups, thoughtful fluid administration earlier during the course of illness decreased mortality, AKI incidence, and need for RRT in comparison to historical controls. This time-sensitive nature of shock resuscitation has been stressed upon many times due to evidence of higher mortality in children with a delay in reversal of shock (85–88).

A number of studies, however, have questioned the efficacy of EGDT. Three multicenter RCTs- ProCESS, ProMISE and ARISE, did not show a statistically significant reduction in 90d mortality with EGDT but reported mortality was lower than the anticipated rate (89–91). Current surviving sepsis campaign guidelines (SSCG) do not endorse EGDT but still permit the use of previous targets as no harm was documented in these trials (92). Subsequent meta-analyses have yielded inconclusive results and equipoise still exists (93). Nevertheless, recruitment of less sick patients, treatment crossover due to non-blinding, and early institution of aggressive antibiotic therapy could haves biased the results of these negative trials (94).

Workman and colleagues recently showed that compliance with SSCG was not associated with better outcomes compared with usual therapy, which was administered more slowly in the emergency department (95). Since all children were treated rapidly, they reported low morbidity and mortality, and underscored the importance of rapid recognition and treatment of septic shock.

## Fluids: type in critically ill children?

In children with diabetic ketoacidosis, NS (normal saline) is the preferred resuscitating fluid but development of significant hyperchloremic acidosis necessitates a switch to hypotonic fluids such as 1/2NS or more physiological choices with lower chloride but high sodium concentrations such as LR (lactated Ringer's), Hartmann's solution, or plasmalyte (96). In pediatric acute respiratory distress syndrome, the PALICC recommends monitoring and titrating fluid balance to maintain adequate intravascular volume, while aiming to prevent positive fluid balance (97). Mechanical ventilation in acute lung injury reduces the amount of maintenance fluids, so in these patients the preferred choice is a dextrose containing solution with NS (or one of the other more physiological solutions) at two-thirds of the maintenance rate that is used recommended for non-mechanically ventilated patients (96).

In cases of pediatric sepsis, different studies have shown varying and conflicting data on which fluids provide adequate resuscitation without increasing mortality. The SSCG recommend crystalloids as the initial resuscitation fluid of choice, with addition of albumin only if substantial amounts of crystalloids are required (98). However, UK investigators obtained outstanding results with use of albumin boluses in resuscitation of children with meningococcal septic shock (99). In an RCT, Maitland et al. demonstrated a mortality benefit in malarial septic shock with albumin when compared to crystalloids (100). Nevertheless, evidence strongly recommends against using hydroxyethyl starch in adults and children due to an acute increase in RRT and red cell transfusion requirement (92, 98).

In a recent large matched cohort study on pediatric sepsis, LR resuscitation was not associated with reduced mortality, AKI, or dialysis even when matched by fluid volume and proportionate LR utilization (101). Mortality increased with larger fluid volumes and decreased with a greater proportion of fluid given as LR, but there were no differences between LR and NS groups after matching within volume quartiles, by proportionate LR utilization, or in the separate matched analysis of LR-only patients. However, LR was preferentially used either as first-line fluid in patients with lower illness severity or as an adjunctive fluid in patients who received large amounts of fluid, and the matching algorithm was least effective in the most severely ill patients receiving the largest total fluid volumes. This could have masked a true benefit of LR. Further studies and well-designed RCTs are needed to clarify the optimal type and dose of resuscitation fluid.

## Management of fluid overload

Early FO has garnered much attention as it seems to be a more decisive factor than late FO in predicting survival because the peak of illness is typically in first few days post-admission and the CFB curve generally flattens out after this period (58, 59, 71, 75, 76). Hence early fluid resuscitation is most likely beneficial, whereas early FO is not. Many studies advocate use of standard concentration drug infusion to prevent iatrogenic FO because many drugs are delivered as dilute solutions which significantly add to the daily fluid intake. This effect can be drastic in infants who are unnecessarily exposed to excess fluid through weight-based infusions (102). The concerns about perioperative hyponatremia in children with use of hypotonic maintenance fluids have been addressed lately via a switch to isotonic fluids (96, 103, 104).

The lack of a specific recommendation on optimal timing of RRT institution still plagues clinicians. CRRT remains the modality of choice in pediatric critical care. The American College of Critical Care Medicine recommends that a threshold of 10% should be construed as the time for intervention (14). An expert discussion of the pharmacologic management of FO in critically ill patients was recently published (105). Furosemide effectively achieves diuresis in critically ill children, but RCTs have failed to demonstrate any benefit of diuretics in preventing or treating AKI (106, 107). The furosemide stress test can be employed to assess the response to diuretics as resistance only delays RRT initiation (108). Aminophylline and fenoldopam have received medical attention owing to their potential in ameliorating AKI. The KDIGO guidelines recommend the use of a single dose of prophylactic theophylline in infants with severe perinatal asphyxia who are vulnerable to develop AKI (109). Use of these agents in post-cardiac surgery infants has also resulted in an improved excretory function and a reduction in urinary AKI biomarkers (110, 111).

## One size does not fit all – is it worthwhile to predict fluid responsiveness?

The prediction of FR (fluid responsiveness) in critically ill children remains largely unembraced to date despite its potential role in obviating FO as only 40–60 % of patients typically respond to fluids by increasing the cardiac output by more than 10–15% (112). The hemodynamic predictor variables have been classified as static and dynamic. Static variables are volumetric and include preload measures based on a single observation in time, namely maximal end diastolic volume, CVP or pulmonary artery occlusion pressure. Dynamic variables consider the cyclical changes in preload occurring during ventilation which are apparent as changes in stroke volume via the Frank-Starling law. These include systolic pressure variation (SPV), pulse pressure variation (PPV), and stroke volume variation (SVV). Respiratory variation in aortic blood flow peak velocity has been shown to be the only variable predictive of FR in children (112, 113). A recent systematic review demonstrated the limited predictive ability of static and dynamic variables in pediatric patients (112). Static variables did not predict FR in children, similar to the evidence in adults. However, dynamic variables based on arterial blood pressure also did not predict FR unlike adults. This probably could be due to age-related differences in vascular mechanical properties which affect the arterial waveform behavior. Evidence for dynamic variables based on plethysmography was deemed inconclusive. The insensitivity of these variables can be ascribed to numerous reasons, namely low tidal volume ventilation, spontaneous respirations, arrhythmias and valvular regurgitation. Inclusion of baseline contractility may improve the predictive potential of these variables. Standardized volume loading (e.g., 10 ml/kg) may not lead to consistent changes in ventricular EDV due to inter-individual variance (114). There is a need to clarify how differences in lung, vascular, and cardiac compliances affect predictive potential of dynamic variables in children as volume loading should be tailored for only those patients who are likely to increase stroke volume.

## Limitations

Literature on fluid resuscitation in pediatric patients is vast but there is no general consensus on the type and dose of resuscitation fluid. Though there is an association between fluid overload and the risk of BPD in a neonate, there is no consensus or guideline on whether fluid restriction or the management of FO will reduce BPD incidence. This is just one example showing the current lack of knowledge on fluid management in children. Further studies and well-designed RCTs are necessary to provide physicians with optimal guidelines. The limited predictability of fluid responsiveness in children along with limited knowledge on the utility of early goal directed therapy further complicates the management of FO. There is a need for more studies to better clarify the effects of FO and create a standardized set of guidelines to assist physicians with management and decrease adverse outcomes.

## Conclusions

Managing fluid overload in a critically ill pediatric patient is no simple task and poses a multitude of complications. To begin with, the lack of a proper consensus on a method for calculating FO due to the significant number of uncertainties in estimating intake and output makes management a challenge. While FO itself is not a direct marker of mortality, the adverse effects of FO lead to patients being vulnerable to an increased risk of morbidity and mortality. FO puts patients at a risk of being underdiagnosed with AKI and delays treatment, raises odds for mortality associated with CCRT complications, can lead to increased hospital and ICU stay, and prolonged ventilator support in the critically ill population. Given the current lack of knowledge on fluid management in children, further studies and RCTs are necessary to provide guidelines to physicians.

## Author contributions

All authors listed have made a substantial, direct and intellectual contribution to the work, and approved it for publication. RR, SKS, NW and SBB conceptualized the study. RR, SKS, NW and SBB wrote the review. MV and VK made significant contributions in writing, editing and revising the study.

### Conflict of interest statement

The authors declare that the research was conducted in the absence of any commercial or financial relationships that could be construed as a potential conflict of interest.
